# Use of Hepatitis B-e Antigen to Identify Pregnant Women With Hepatitis B Virus Infection Who Need Antiviral Therapy for Prevention of Mother-to-child Transmission

**DOI:** 10.7759/cureus.18430

**Published:** 2021-10-01

**Authors:** Chanya Jiragraivutidej, Pisit Tangkijvanich, Surasith Chaithongwongwatthana

**Affiliations:** 1 Department of Obstetrics and Gynecology, Faculty of Medicine, Chulalongkorn University, Bangkok, THA; 2 Department of Obstetrics and Gynecology, Bangkok Metropolitan Administration General Hospital, Bangkok, THA; 3 Department of Biochemistry, Faculty of Medicine, Chulalongkorn University, Bangkok, THA; 4 Center of Excellence in Hepatitis and Liver Cancer, Faculty of Medicine, Chulalongkorn University, Bangkok, THA

**Keywords:** hepatitis b-e antigen, antiviral therapy, viral load, hepatitis b, mother-to-child transmission

## Abstract

Objective

To evaluate the performance of hepatitis B-e antigen (HBeAg) for identifying pregnant women infected with hepatitis B virus (HBV) who are having a high viral load.

Methods

A cross-sectional study was conducted at the tertiary care hospital in Bangkok, Thailand between August 2017 and August 2018. Ninety-six pregnant women having positive hepatitis B-s antigen (HBsAg) results were invited to participate into the study. Clinical data and blood samples were collected and tested for HBeAg and HBV DNA levels. Data were reported as percentage and 95% confidence interval (CI).

Results

High viral load was found in 25 women (26.0%, 95% CI: 18.3% to 35.6%) and HBeAg showed positive results in 33 women (34.4%, 95% CI: 25.6% to 44.3%). Among antiviral-naïve women, 24 of 30 cases having positive HBeAg results had high viral load (80.0%, 95% CI: 62.7% to 90.5%) while only 1 of 62 negative HBeAg women had high viral load (1.6%, 95% CI: 0.3% to 8.6%).

Conclusion

About one-fourth of HBV-infected pregnant women were at high risk for mother-to-child transmission (MTCT) of the virus and needed antiviral drugs for reducing MTCT. HBeAg may be used to identify women at high risk for MTCT of HBV in a low-resource setting where HBV DNA level test is not available.

## Introduction

Hepatitis B virus (HBV) infection is a major global health problem. In 2015, 257 million people worldwide were estimated to be chronically HBV-infected and almost 900,000 deaths resulted from long-term complications of chronic HBV infection including cirrhosis and hepatocellular carcinoma [1]. The World Health Organization has set a global health sector strategy on viral hepatitis to achieve the goal of eliminating viral hepatitis as a major public health threat by 2030 [2]. Prevention of mother-to-child transmission (MTCT) of HBV is a key intervention to eradicate newly infected neonates that are at high risk for developing chronic infection [3]. MTCT of HBV can be prevented by immunoprophylaxis strategies including administration of infant universal vaccination and passive immunization with hepatitis B immunoglobulin (HBIG) for infants from mothers having chronic HBV infection [3]. However, immunoprophylaxis failure was found in about 5-15% of infants and is more likely to occur in mothers testing positive for hepatitis B-e antigen (HBeAg) and/or high viral load or HBV DNA level [4,5]. There is growing evidence of an antepartum antiviral drug in reducing MTCT of HBV in high viral load pregnant women [6-10]. In 2016, the American Association for the Study of Liver Diseases (AASLD) recommended providing antiviral therapy in HBV-infected pregnant women with an HBV DNA level >200,000 IU/ml [11]. Antivirals used in pregnant women to reduce the risk of MTCT of HBV include lamivudine, telbivudine, and tenofovir disoproxil fumarate (TDF). It is suggested that these antivirals are started at 28-32 weeks of gestation and discontinued at birth to three months postpartum [11].

Lack of availability and high cost for HBV DNA level tests are major limitations to introduce antivirals for MTCT prevention of HBV in the public health services in Thailand [12]. Compared to measuring a viral load, an HBeAg test seems more available and affordable. The use of HBeAg instead of HBV DNA level as an indicator for antiviral therapy has been proposed in resource-limited areas. However, this strategy may result in over-treated or under-treated cases. The present study aimed to evaluate the performance of HBeAg for identifying pregnant women infected with hepatitis B virus who have a high viral load.

## Materials and methods

The present study was a cross-sectional descriptive study conducted at the King Chulalongkorn Memorial Hospital (KCMH), Bangkok, Thailand between August 2017 and August 2018. The study was reviewed and approved by the Institutional Review Board of the Faculty of Medicine, Chulalongkorn University (IRB Number 129/60) and conducted in accordance with approved guidelines. Written informed consent was obtained from each participant prior to the study enrollment. The sample size calculation for estimating the infinite population proportion shown 90 participants were required when the prevalence of HBeAg was expected to be 30% with a precision of 10% for 95% level of confidence and 10% drop-out rate.

The study population were pregnant women with HBV infection at the antenatal clinic, delivery room, or obstetrics ward at KCMH. Inclusion criteria were Thai pregnant women aged over 18 years with positive hepatitis B-s antigen (HBsAg) results. Exclusion criteria were women taking immunosuppressive drugs or agents; having autoimmune diseases or being immunodeficient. Every pregnant woman attending the antenatal clinic or admitted to KCMH was routinely screened for HBV, human immunodeficiency virus (HIV), and syphilis infections at the first visit if she had never been tested during the current pregnancy. Women with HBsAg-positive results were approached and gave informed consent to collect blood samples for HBeAg, HBV DNA, alanine aminotransferase (ALT), aspartate aminotransferase (AST), and creatinine levels. Past medical history, current medications, previous clinical symptoms, diagnosis or treatment of hepatitis, and family history of HBV infection were recorded. If participants were found to have an indication for antiviral treatment, consultation with the gastroenterologist was appointed and the treatment was started accordingly.

High viral load in the present study is defined as HBV DNA level >200,000 IU/ml according to the antiviral threshold of pregnant women at high risk for MTCT of HBV [11]. The upper limit of normal (ULN) for ALT level is defined as 19 U/L for women [11]. TDF were prescribed to women who had a high viral load to prevent MTCT of HBV. Women who had an elevation of ALT >2 ULN plus HBV DNA level >2,000 IU/ml (HBeAg-negative) or HBV DNA level >20,000 IU/ml (HBeAg-positive) were indicated for antiviral treatment [11].

All blood samples collected were submitted and processed at the central, microbiology, and immunology laboratories at KCMH. HBV DNA level was measured with real-time PCR technique for quantification of HBV using the Abbott RealTime HBV Viral Load Assay (Abbott Diagnostics Inc., Abbott Park, IL, USA) [13]. A chemiluminescent microparticle immunoassay (CMIA) was used for the determination of HBeAg status using the Architect i2000 analyzer (Abbott Diagnostics, Inc.) [14].

The main study outcomes were the percentages of HBV-infected pregnant women having HBV DNA levels>200,000 IU/ml and of those having HBeAg-positive results. HBeAg status of pregnant women in low-risk and high-risk for MTCT of HBV was explored. Data were reported using descriptive statistics including percentage and 95% confidence interval (CI) for categorical variables. Continuous variables were described as mean with standard deviation (SD) or median with interquartile range (IQR) when appropriate. All statistical analyses were performed using SPSS software version 22.0 (IBM Corp., Armonk NY, USA) [15].

## Results

Ninety-six pregnant women were eligible and recruited in the present study. Baseline characteristics are shown in Table 1. The mean age of the participants was 33.1 years and the mean gestational age at blood sample collection was 28.8 weeks. Most of the women (84 cases, 87.5%) had ALT and AST of less than 2 ULN.

**Table 1:** Baseline characteristics of HBV-infected pregnant women ALT: alanine aminotransferase, AST: aspartate aminotransferase, IQR: interquartile range, SD: standard deviation

Parameters	N= 96
Age (years), mean ± SD	33.1 ± 4.3
Gestational age (weeks), mean ± SD	28.8 ± 8.9
Gravidity, n (%)	
1	39 (40.6%)
2	36 (37.5%)
≥3	21 (21.9%)
ALT (U/L), median (IQR)	18 (13 to 26)
AST (U/L), median (IQR)	20 (16 to 28)
Creatinine (mg/dL), mean ± SD	0.55 ± 0.1
Platelets (×10^3^/µL), mean ± SD	241 ± 54

Twenty-five women (26.0%, 95% CI 18.3% to 35.6%) had HBV DNA level >200,000 IU/ml (Table 2). HBeAg results were positive in 33 cases (34.4%, 95% CI 25.6% to 44.3%). All four women who underwent antiviral therapy before pregnancy were found to have HBV DNA levels <2,000 IU/ml, but only one case had an HBeAg-negative result.

**Table 2:** HBV infection profiles of pregnant women (N = 96) ^a^Elevated ALT + (HBV DNA>2,000 in negative HBeAg) or elevated ALT + (HBV DNA>20,000 in positive HBeAg) ^b^ALT ≤2 ULN, but HBV DNA level >200,000 IU/ml ALT: alanine aminotransferase, ULN: upper limit of normal, MTCT: mother-to-child transmission

Parameters	N (%)
Elevated ALT (>2 ULN)	12 (12.5)
Positive HBeAg	33 (34.4)
HBV DNA level (IU/ml)	
≤2,000	57 (59.4)
2,001–20,000	11 (11.5)
20,001–200,000	3 (3.1)
>200,000	25 (26.0)
Need antiviral therapy	
Total	31 (32.3)
On antiviral before pregnancy	4 (4.2)
Indicated for treatment^a^	9 (9.4)
For only preventing MTCT of HBV^b^	18 (18.7)

Among antiviral-naïve women, a high viral load was found in 24 of 30 women with HBeAg-positive results (80.0%, 95% CI 62.7% to 90.5%). Of 62 HBeAg-negative result women, there was only one case (1.6%, 95% CI 0.3% to 8.6%) who had high HBV DNA levels (Table 3).

**Table 3:** Comparison of HBV infection profiles between cases with HBeAg negative and cases with HBeAg positive among antiviral-naïve women (N = 92) ^a^Elevated ALT + (HBV DNA>2,000 in negative HBeAg) or elevated ALT + (HBV DNA>20,000 in positive HBeAg) ^b^ALT ≤2 ULN, but HBV DNA level >200,000 IU/ml ALT: alanine aminotransferase, ULN: upper limit of normal, MTCT: mother-to-child transmission

Parameters	Negative HBeAg (N = 62)	Positive HBeAg (N = 30)
HBV DNA >200,000 IU/ml	1 (1.6%)	24 (80.0%)
Elevated ALT (>2 ULN)	2 (3.2%)	9 (30.0%)
Need antiviral therapy		
Total	2 (3.2%)	25 (83.3%)
Indicated for treatment^a^	1 (1.6%)	8 (26.7%)
For only preventing MTCT of HBV^b^	1 (1.6%)	17 (56.6%)

## Discussion

The present study demonstrated that 26% of HBV-infected pregnant women had HBV DNA levels >200,000 IU/ml which increases the risk for MTCT and immunoprophylaxis failure. Antiviral therapy during the third trimester of pregnancy was found to be highly effective for the prevention of MTCT in these high viral load mothers [6-10]. Although this intervention has been recommended for a period of time now [11], there are still some limitations with its implementation.

In 2015, 2.2-3 million people in Thailand were estimated to be chronic HBV-infected, mostly those who were born prior to 1992 when universal HBV vaccination in newborns was first implemented in an expanded nationwide immunization program [16,17]. Since the prevalence of chronic HBV infection among reproductive-aged people is estimated at 3-6% [16,17], there are 20,000-40,000 Thai pregnant women with chronic HBV infection annually. If immunoprophylaxis failure occurs in 5% of this population, at least 1,000 neonates will be HBV-infected and at risk for developing chronic infection and with severe consequences later on. The number of newly infected newborns might be higher at border areas where high HBV seroprevalence among pregnant women is reported [18].

Antiviral therapy for the prevention of MTCT of HBV is indicated when maternal HBV DNA levels are more than 200,000 IU/ml. The major limitations of this strategy are the availability and cost of the HBV DNA level test. One HBV DNA level test at KCMH is approximately 2,000 Baht (65 US$). It is estimated that each year, more than 40 million Baht (1.3 million US$) would be needed to test all Thai HBV-infected pregnant women. When comparing HBV DNA level test to HBeAg test and antiviral drugs, the latter two are more readily available and affordable. HBeAg is associated with active viral replication and high viral load [19]. Women having HBeAg-positive results were found in 34.4% of all participants which is similar to those reported in previous studies [18,19]. According to our study, HBeAg status was associated with high HBV DNA levels when excluding women who were already taking antiviral therapy. Among antiviral-naïve women, over-treatment would be at 16.7% if HBeAg-positive results were used to provide antiviral treatment for pregnant women while only 1.6% of high viral load patients would be missed in women with HBeAg-negative results. A study using a mathematical model reported that HBeAg and viral load testing in pregnant women with HBV infection to determine the need for antiviral therapy were both cost-effective [20]. However, a recent clinical trial [21] did not find a significant difference in terms of transmission rate between TDF and placebo groups in women with HBeAg-positive results.

Nine women in the present study who were in the immunoactive phase of HBV infection had both elevated ALT and viral load and were indicated for antiviral therapy [11]. Seven of them had a viral load of more than 200,000 IU/mL, while the others had a low viral load (HBeAg-negative with HBV DNA 3,866 IU/L and HBeAg-positive with HBV DNA 43,296 IU/L).

There were some limitations of the present study. First, the study population was made up of only pregnant women at KCMH, which may not be generalized to the rest of the women in the country. Second, the variation in gestational age at blood collection could have accounted for some inconsistencies in our findings because our participants were recruited at different gestational ages due to many factors including time of patient identification, the decision to join the study, and other preferences. Nonetheless, a previous study demonstrated 97.8% consistency of HBV DNA levels between two time points during pregnancy [22].

## Conclusions

About one-fourth of HBV-infected pregnant women have a high risk for MTCT of HBV, often requiring antiviral therapy. The present study supports the use of HBeAg as an indicator for the administration of antiviral agents to prevent MTCT instead of HBV DNA level in limited-resource settings. HBeAg might be useful as triage for selecting case-appropriate HBV DNA level tests. Future larger-scale studies for national implication and further innovative research to develop more cost-effective tests to identify high viral load mothers are needed.
